# Innovative strategies for managing hallucinations by exploring effects of tDCS on source monitoring abilities

**DOI:** 10.1038/s41598-024-67279-0

**Published:** 2024-07-17

**Authors:** Gaurav Sharma, Vinay Chitturi, Vivek Kumar Sharma, Rajesh Kathrotia, Pradip Barde, Naresh Parmar, Medhavi Sharma, Ragini D. Singh

**Affiliations:** 1 Department of Physiology, All India Institute of Medical Sciences, Rajkot, Gujarat India; 2 Department of Obstetrics and Gynaecology, All India Institute of Medical Sciences, Rajkot, Gujarat India; 3 Department of Biochemistry, All India Institute of Medical Sciences, Rajkot, Gujarat India

**Keywords:** Source monitoring, Auditory hallucinations, Neuromodulation, Transcranial direct current stimulation, Neuroscience, Physiology, Neurology

## Abstract

This randomised, crossover, sham-controlled study explored the neural basis of source-monitoring, a crucial cognitive process implicated in schizophrenia. Left superior temporal gyrus (STG) and dorsolateral prefrontal cortex (DLPFC) were the key focus areas. Thirty participants without neurological or psychological disorders underwent offline sham and active tDCS sessions with specific electrode montage targeting the left STG and DLPFC. Source-monitoring tasks, reality monitoring (Hear-Imagine), internal source-monitoring (Say-Imagine), and external source monitoring (Virtual–Real) were administered. Paired t-test and estimation statistics was performed with Graphpad version 10.1.0. The Benjamini–Hochberg procedure was employed to control the false discovery rate in multiple hypothesis testing. A significant improvement in internal source monitoring tasks (p = 0.001, Cohen's d = 0.97) was observed, but reality monitoring tasks demonstrated moderate improvement (p = 0.02, Cohen's d = 0.44). The study provides insights into the neural mechanisms of source monitoring in healthy individuals and proposes tDCS as a therapeutic intervention, laying the foundation for future studies to refine tDCS protocols and develop individualized approaches to address source monitoring deficits in schizophrenia.

## Introduction

Schizophrenia is a mental disorder that typically emerges during late adolescence or early adulthood^1^ and affects approximately 23.6 million people worldwide, with new cases increasing from 941,000 to 1.3 million between 1990 and 2019^2^. Despite ongoing efforts, treatment outcomes using best practices often do not meet the desired standards, with only 13.5% of individuals achieving both clinical and social recovery^2^. Schizophrenia can manifest with hallucinations, with auditory hallucinations being the most common, affecting approximately 70% of cases^3^. Interestingly, visual hallucinations occur in half of these cases. Other hallucinations, such as tactile and olfactory hallucinations, are less common^4^. It is worth mentioning that source monitoring deficit is closely linked to schizophrenia and other psychotic disorders, which are characterized by difficulties in distinguishing between external reality and internal experiences, leading to hallucinations and delusions^4^.

Depending on the source of the information, source monitoring can be categorized as external source monitoring (i.e., distinguishing between two or more external sources), internal source monitoring (i.e., distinguishing between two or more internal sources), and reality monitoring (i.e., distinguishing between external and internal sources). Source monitoring is a fundamental component of human cognition, significantly contributing to our ability to navigate and understand the complex interplay between memory and reality, as documented in the scientific literature^4–6^.

In the field of cognitive science, various pathological conditions may often result in delusional and hallucinatory experiences owing to disruptions in the source-monitoring process. Specifically, misattribution of inner speech to an external source is suggested to be closely linked to the emergence of auditory verbal hallucinations in individuals with schizophrenia^7,8^. In the context of schizophrenia, individuals may tend to misconstrue their inner dialogue as coming from an external source and attribute their imaginative thoughts as spoken words^8,9^. These perceptual phenomena are suggested to be rooted in the vivid nature of mental imagery^10^ but are erroneously attributed to external stimuli^4^.

The left superior temporal gyrus (STG) has been implicated in the facilitation of intense mental imagery and manifestation of audio-visual hallucinations^11,12^. Furthermore, it is suggested to contribute to the genesis of reality monitoring errors, where internal thoughts are inaccurately attributed to verbal stimuli originating from an external source, thereby giving rise to auditory and visual hallucinatory experiences^13^. Notably, previous research has shown increased activity within the STG when individuals are exposed to auditory stimuli from external sources, whereas this neural response is notably absent during instances of inner speech generation^14,15^. Importantly, reduced activation within the prefrontal cortex has been empirically associated with the occurrence of source monitoring errors^15^.

Mondino et al.^16^ conducted a notable study in which they applied transcranial direct current stimulation (tDCS) to individuals diagnosed with schizophrenia. This study demonstrated a significant reduction in source-monitoring errors among these individuals. In tDCS treatment, the cathodal electrode was positioned over the left STG, while the anodal electrode was placed over the left dorsolateral prefrontal cortex (DLPFC). The basis for this intervention is rooted in the idea that auditory verbal hallucinations (AVH) and, by extension, source monitoring errors, are linked to the overactivity of regions involved in speech perception, particularly the temporo-parietal region. Simultaneously, there is reduced activity in the prefrontal areas responsible for cognitive control processes^17–19^. In this approach, the cathodal electrode aims to reduce hyperactivity in speech perception regions, while the anodal electrode works to enhance activity in cognitive control areas^19^. The findings of Mondino's study^16^ provide further support for the involvement of the STG and DLPFC in processes governing source monitoring.

In a study on schizophrenia patients with medication-resistant auditory verbal hallucinations, those receiving active tDCS with anode placed over the left dorsolateral prefrontal cortex and the cathode over the left temporo-parietal cortex for 20 min twice daily showed a significant 31% reduction in hallucinations compared to sham stimulation.^19^.

Mondino et al.^20^ conducted a study to investigate the effects of tDCS on source monitoring in healthy individuals. They examined two brain regions separately, the left STG and DLPFC, using anodal and cathodal tDCS, respectively. To ensure precise stimulation, a larger reference electrode was strategically placed on the right occipital cortex to reap the beneficial effects of weak currents on brain activity.

The study revealed that anodal tDCS applied to the left temporo-parietal junction (TPJ), a component of the STG, increased the tendency to attribute internally generated speech to externally perceived speech, known as reality monitoring. However, it had no noticeable impact on internal source monitoring, suggesting that this intervention primarily affected the reality monitoring.

Conversely, cathodal tDCS over the left prefrontal cortex (PFC) did not show any discernible modulation of source monitoring, including both internal source and reality monitoring^20^. Notably, the hypertemporal/hypofrontal model used in a therapeutic study by Mondino et al.^16^ was not directly tested in this study because the left STG and left DLPFC were stimulated in isolation.

In a separate study, Moseley et al.^21^ explored the role of the right anterior medial Prefrontal Cortex (PFC) in conjunction with tDCS. Participants engaged in a reality monitoring task during three distinct sessions: one with active tDCS, where the cathode was over the right medial PFC and the anode over the left STG; one with sham tDCS (the same electrode placement but with no active stimulation); and another with active tDCS using a different electrode montage, with the cathode on the right medial PFC and the anode on the left visual area, serving as a control site. Two discrete experiments were conducted in this comprehensive investigation, each involving a different temporal stage of the cognitive task. In the initial experiment, tDCS was administered during the encoding phase, a juncture at which participants were tasked to memorize a list of words. The subsequent experiment involved the application of tDCS during the testing phase, wherein participants were required to discriminate whether a presented word had been heard previously or merely imagined.The outcomes of these experiments did not reveal any discernible impact of tDCS in comparison with sham tDCS on the process of reality monitoring.

While it is noteworthy that some authors have postulated that cathodal tDCS seldom elicits inhibitory effects in cognitive tasks^22,23^, the observed disparities in results between the studies conducted by Mondino et al.^20^ and Moseley et al.^21^ may potentially be attributed to variations in current intensity, electrode montage configuration (specifically, the distinction between ipsilateral and contralateral hemisphere placements), or temporal alignment of tDCS administration (i.e., whether it coincided with the encoding or testing phase of the task).

Collectively, examinations involving both healthy individuals and individuals afflicted with AVHs have illuminated the crucial role of the PFC and STG in the intricate processes of source monitoring. These neural regions, integral to the AVH manifestation, also demonstrate significance in source monitoring. Nevertheless, ongoing endeavors aimed at mitigating AVHs^24^ while concurrently enhancing source-monitoring capacities through tDCS^16^ are somewhat deficient in elucidating the precise neural mechanisms underpinning the electrode montage employed in these therapeutic investigations. Furthermore, within the realm of neuroimaging studies conducted on individuals afflicted by schizophrenia and AVHs, investigations predominantly concentrate on source monitoring in a broad context without discerning the nuances between reality monitoring and internal source monitoring^6^. Intriguingly, investigations utilizing tDCS have produced inconsistent findings when discriminating between these two distinct cognitive processes^20,21^. Consequently, our research endeavors were directed towards direct examination of the hypertemporal/hypofrontal model. This entailed positioning the anode over the left STG and cathode over the left DLPFC in healthy participants. This electrode configuration emulated the presumed neural activity pattern underlying both AVHs and source monitoring deficits observed in patients with schizophrenia. We hypothesized that the active tDCS would yield more robust behavioral effects on source monitoring capacities in the active experiment compared to the sham experiment. This assumption is grounded in the understanding that active tDCS has a distinct impact on neural activity and behavioral outcomes, as supported by prior research^24^.

While formulating our study design, we encountered a study akin to ours, where tDCS was found to enhance internal source monitoring abilities in healthy participants with same montages^25^. This study shared similarities with ours, but a pivotal distinction was observed. They compared Offline tDCS, where active tDCS was applied and discontinued before task execution, against Online tDCS, where active tDCS was applied and sustained during task performance. Notably, the investigation delved into the post-effects of tDCS. In the offline experiment, participants underwent fMRI, while the online counterpart took place in a distinct setting. Additionally, their study was not a crossover study, as the online and offline experiment was conducted on different participants. While the referenced study primarily focused on the on–off effects and after-effects of tDCS, the aim of our study was to test the hypertemporal/hypofrontal model directly by placing the anode over the left STG and the cathode over the left DLPFC on source monitoring abilities (Hear-Imagine, Say-Imagine, and Real-Virtual) in healthy individuals. Hence, we set to explore tDCS as a potential effect of sham vs active tDCS on various types of source monitoring abilities in same environment for the same subjects.

In our study, we conducted two sets of experiments : the first set involved a sham experiment, where source-monitoring assessments were carried out following sham tDCS stimulation. The second set comprised an active experiment, in which source-monitoring tasks took place after the application of active tDCS. The inclusion of sham tDCS was primarily exploratory, while the active tDCS was to study the effects of tDCS on the source monitoring abilities.

Individuals with schizophrenia exhibit inferior performance on source-monitoring tasks, which involve making judgments about the origins of memories, knowledge, and beliefs. While source monitoring is recognized as a significant cognitive bias associated with reality distortions and psychotic symptoms, our study's primary objective was to explore the effects of tDCS various types of source monitoring abilities and to understand the neurocognitive mechanisms underlying the relationship between source monitoring and neuropsychological functioning.

## Materials and methods

### Participants

This randomised, crossover, sham-controlled study recruited 30 healthy participants. We used ‘word of mouth’ and the ‘snowball’ sampling technique to recruit participants for the study. The exclusion criteria were history or current diagnosis of neurological or psychological disorders, head trauma, metallic implants, migraines, epilepsy in the family, pregnancy, recent substance use, or severe skin diseases in the electrode placement area. Additional exclusion details can be found in ‘Supplement A’. This study was conducted in accordance with the Declaration of Helsinki and the current ethical codes. Informed consent was obtained from all the participants. The study protocol was approved by the Institutional Ethics Committee of All India Institute of Medical Sciences, Rajkot, Gujarat, India (Approval No: IEC/20/2022).

### Questionnaires

We collected basic demographic data from all the participants, including age, sex, place of residence, and education level. Additionally, we used the Edinburgh Handedness Inventory to determine the participants' handedness^26^. This inventory provides a score ranging from -100 (exclusive left-handedness) to + 100 (exclusive right-handedness). Notably, prior research^27^ has indicated that approximately 70% of left-handed individuals show language specialization in the left hemisphere of the brain compared to 95% of right-handed individuals. Hence, for this study, we exclusively included right-handed participants, excluding those with scores between – 50 and + 50.

This study involved source-monitoring tasks that assessed reality monitoring, internal source monitoring, and external source monitoring. These tasks were based on the previous studies by Keefe et al.^9^ and Brunelin et al.^7^. Emotionally neutral words in English were used for these tasks, with positive and negative words excluded because of differing emotional processing effects^28^. The auditory stimuli for the Hear-Imagine task were computer-generated using a male voice with a native Indian accent. To prevent learning effects between tDCS sessions, two separate set of words were created for each sham and active tDCS tasks (Volume 1 and Volume 2) and either Volume 1 or Volume 2 was assigned randomly to sham and active tDCS. Each Volume had with three versions (Version A, B, and C) based on the sequence of tasks of 30 words each. The order of tasks (‘Hear-Imagine, Say-Imagine, and Virtual-Real’) was assigned randomly as per the version A, B or C assigned. Each task consisted of a training phase and a test phase. In the test phase, 16 words were displayed on a computer screen for 5 s, preceded by instructions. A training phase with up to six words explained the task to participants.

In the internal source-monitoring task (‘Say-Imagine’ or SI), participants were instructed to either say the word aloud or imagine saying it when displayed on the computer screen. During the test phase, the participants indicated whether they had spoken the word aloud or imagined doing so. In the external source-monitoring task (‘Virtual-Real’ or RV), participants encountered words from two external sources: prerecorded audio files and a live interviewer. They indicated whether they had heard each word from a computer speaker or a live interviewer during the test phase. In the reality-monitoring task (‘Hear-Imagine’ or HI), the participants were instructed to imagine hearing or actually listen to the presented words on the computer screen. In the test phase, the participants indicated whether they had physically heard the word or imagined its reception. After the presentation of words in all the three tasks, the participant was immediately given a list of 24 words, this list had 16 words from the task and 8 extra words (called distractors) that were not in the task. The participants had to correctly mark the word as per their source (e.g. heard or imagined) or distractor. It was not declared to the participants that there were eight words for each category (heard, imagined, and distractors) to prevent bias and calculations while making response. This study focused on the participants' correct responses as a measure of their cognitive performance and memory. The participants completed the ‘HI’, ‘SI’ and ‘RV’ tasks (in randomized order), and correct responses were carefully recorded. Each source monitoring task (HI, SI, and RV), including both the training and test phases, along with the recall of the sources of 24 words (including distractors), lasted approximately 3 min. A rest period of two minute was provided between each task. In total, the entire experiment took approximately 50 min in the sham session and 50 min in the active tDCS session on separate days. Tasks were conducted immediately after the completion of the tDCS session.

### tDCS

**Figure 1:**
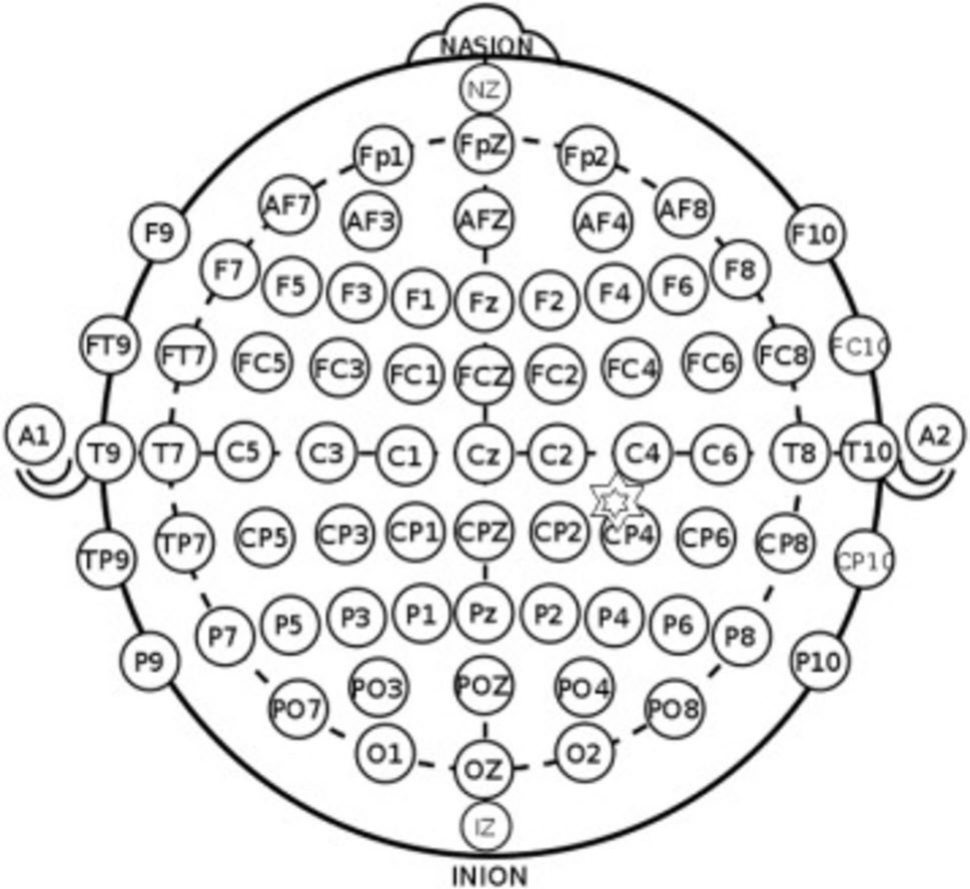
tDCS electrode placement over AF3 and anode placement over CP5.

All participants underwent both sham and active tDCS using a Brain Premium E1 device (Caputron, New York, USA) equipped with 1.5-inches radius circular electrodes. Following the 10/20 system, the electrode was placed at AF3 (left DLPFC), and the anode was placed at CP5 (left STG) (Fig. 1). Normal saline (0.9% saline) was used as a conductive medium to enhance electrical conductivity and reduce impedance, with secure fastening of the electrodes using adjustable rubber bands and Velcro straps.

The tDCS session lasted 20 min at an intensity of 2 mA^29^. During the session of sham or active tDCS, the participants remained seated comfortably and watched nature videos while soothing background music to alleviate any stress or anxiety.

Sham tDCS before active or active tDCS before sham tDCS was assigned randomly but ensuring 15 participants in each arm. This was done at two separate visits, at least 24 h apart but within 3 days. This approach was adopted to account for the potential prolonged effects of a single tDCS session, which may extend to several hours^30^. The cognitive tasks labeled A, B, or C were assigned randomly to the participants with a standardized 2-min interval between each task, during which participants remained seated in their chairs. Strategic placement of the tDCS electrodes was performed for sham trial to as well to ensure proper blinding. The sham procedure involved initiating the tDCS device at 2 mA for a duration of 30 s, followed by a gradual tapering off, ultimately turning off within the subsequent 30 s.

To assess potential side effects of application of tDCS such as headaches and nausea, we used the ‘tDCS Adverse Effects Questionnaire,’ as outlined in Supplement B, before the tDCS session and after completion of source monitoring tasks.

**Figure 2:**
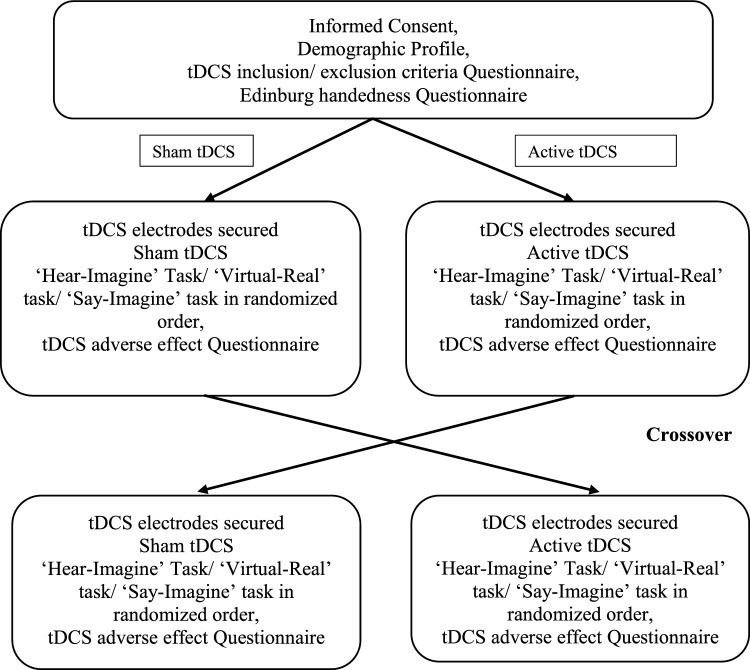
The study flow chart.

The steps involved in this study are briefly outlined in the flowchart (Fig. 2).

### Statistical analysis

Demographics, cognitive performance, and adverse effects of tDCS on source-monitoring errors were thoroughly examined and recorded. Appropriate statistical analysis was performed with software Graph pad prism version 10.2.3 (https://www.graphpad.com/).

### Demographics and adverse effects

Key demographic characteristics, such as age, gender, address, and educational level, were recorded for each participant. Any adverse effects reported by the participants were carefully documented.

### Cognitive performance: comparative analysis of cognitive tasks

Student’s paired sample t-test was used to assess the overall impact of tDCS on source monitoring. The correct responses (as scores) by the participants before and after the tDCS was compared for all the tasks, 'say imagine,' 'hear imagine,' and 'real virtual.'

Statistical significance was set at p < 0.05.

### Evaluation of effect size and precision for practical significance

In addition to significance testing, estimation statistics were performed to focus on the precision and magnitude of the observed effects (effect size) of the tasks. Cohen’s d was used to measure the effect size. It takes the difference between two means and expresses it in standard deviation units. A small effect size (d < 0.2) suggests a minor difference, a medium effect size (0.2 ≤ d < 0.8) indicates a moderate difference, and a large effect size (d ≥ 0.8) signifies a significant divergence between groups. The effect size and precision results in terms of 95% CI were represented as paired estimation plots with a Tufte slope graph. The Tufte slope graph depicts the effect size of the measures.

### Correction for multiple comparisons

The Benjamini–Hochberg (BH) procedure, with a false discovery rate of 0.05, was used to correct for multiple comparisons. Results were deemed significant if the obtained p-value was smaller than the Benjamini–Hochberg critical value.

## Results

### Demographic characteristics

Our cohort consisted of 30 participants, 21 males (70%) and 9 females (30%), with an age range spanning from 20 to 45 years (mean ± SD = 35.50 ± 6.42). The participants held educational qualifications at the graduate level or higher, providing a foundation for their cognitive prowess and analytical aptitude. Furthermore, their competence in English was a crucial consideration, ensuring their ability to complete the study’s cognitive assessments effectively.

Handedness, a potential influencer of task performance, was assessed using the Edinburgh Handedness Inventory. The inclusion of all participants as right-handed, scoring more than 50 on the Edinburgh Handedness Inventory, further ensured the homogeneity of our sample.

### Source monitoring error

#### ‘Say-imagine’ sham vs active-tDCS correct responses

**Figure 3:**
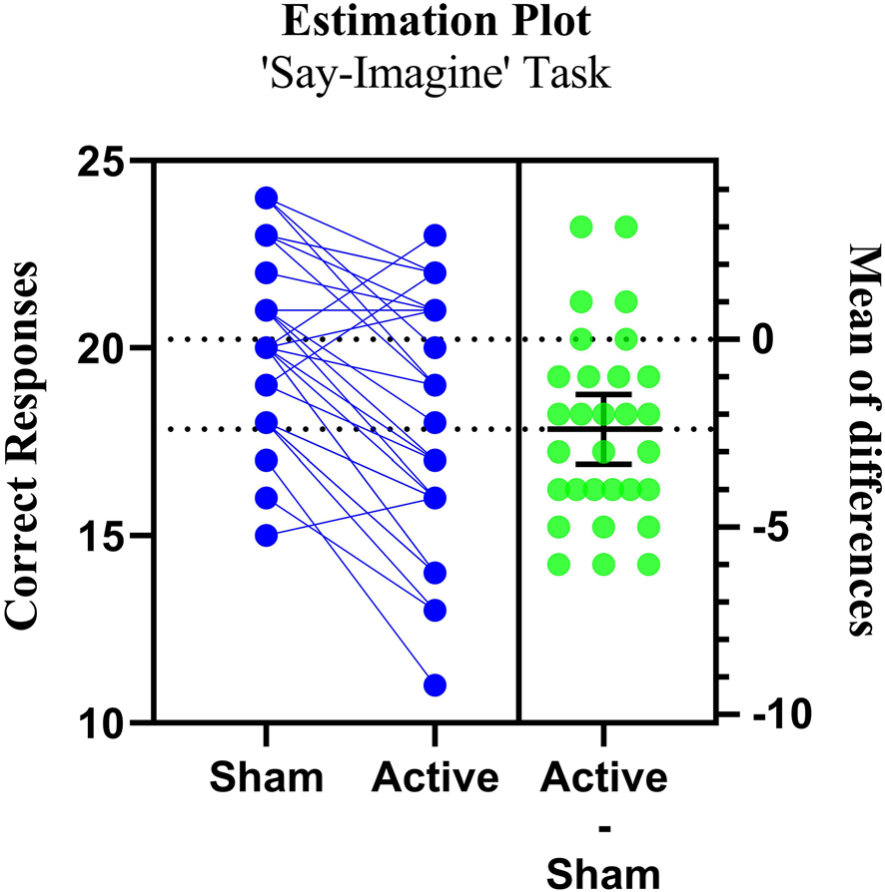
Paired estimation plot depicting the effect size for correct responses in ‘Say-Imagine’ source monitoring task. Raw data (Correct response) from sham and active session for SI task is plotted on the left ‘Y axis’. On the right ‘Y axis’, the mean of differences is plotted along with 95% CI. In this plot, the blue circles represent individual participants. The green circles with error bars represent the mean of each group with a 95% CI.

The paired-sample t-test analysis revealed a significant difference between the sham vs active tDCS sessions (p < 0.001) for ‘SI’ task, accompanied by a substantial Cohen's d of 0.97 (95% CI 0.52–1.40), signifying a large effect size (Table 1). This outcome underscores the considerable influence of the correction intervention on ‘SI’, with active tDCS measures significantly surpassing their sham tDCS measures, both statistically and practically (Fig. 3).

**Table 1:** Sham and active tDCS effects on various source monitoring tasks.

Source monitoring tasks (variable of the study)	N	Mean	SD	t-statistic	df	p value	Benjamini–Hochberg critical value	Cohen’s d	
								Effect size	95% CI
SI correct response sham tDCS	30	20.23	2.37	5.31	29.0	< 0.001	0.016	0.97	0.52 to 1.40
SI correct response active tdcs	30	17.83	3.35						
HI correct response Sham tDCS	30	19.06	2.89	2.41	29.0	0.02	0.033	0.44	0.06 to 0.81
HI correct response Active tDCS	30	17.87	3.26						
RV correct response sham tDCS	30	18.30	3.79	0.98	29.0	0.33	0.05	0.18	−0.18 to 0.54
RV correct response active tDCS	30	17.53	3.52						

*BH* critical value for the Benjamini–Hochberg correction for multiple testing, (i/m) × Q, where i is the rank, m is the total number of tests (m = 3), and Q is the false discovery rate (Q = 0.05). If p value < BH, then p value will be considered significant.

#### ‘Hear-imagine’ sham vs active-tDCS correct responses

**Figure 4:**
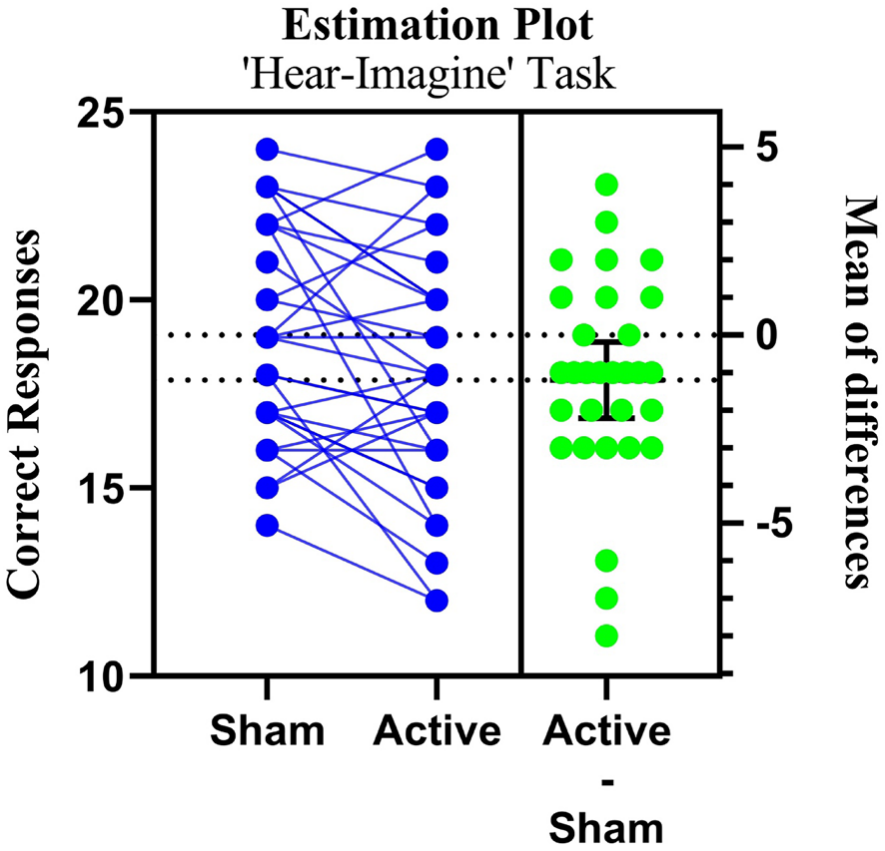
Paired estimation plot depicting the effect size for total correct responses in in ‘Hear-Imagine’ source monitoring task. Raw data (correct response) from sham and active session for HI task is plotted on the left ‘Y axis’. On the right ‘Y axis’, the mean of differences is plotted along with 95% CI. In this plot, the blue circles represent individual participants. The green circles with error bars represent the mean of each group with a 95% CI.

The paired sample t-test for the ‘Hear-Imagine’ task revealed a statistically significant difference in the mean sham and active tDCS (p = 0.02) (Table 1). Although the *p*-value is statistically significant, the effect size of Cohen's d = 0.44 suggests a moderate impact (Fig. 4). Consequently, our correction intervention had a statistically significant effect on ‘HI’, but the effect size was closer to the moderate range.

#### ‘Real-virtual’ sham vs active-tDCS correct responses

**Figure 5:**
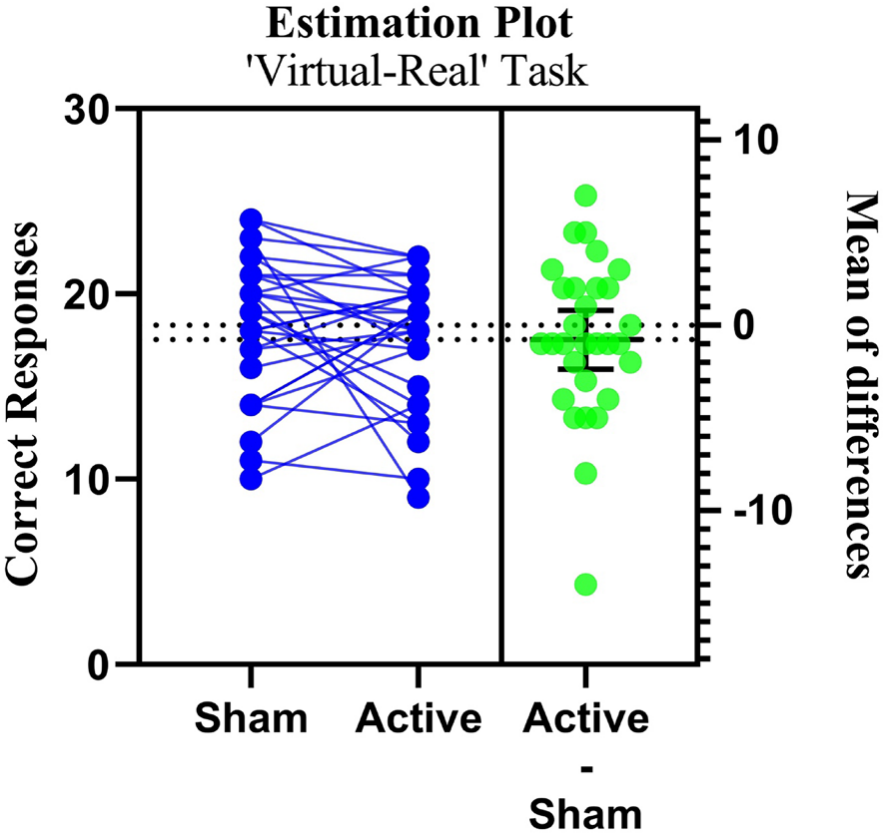
Paired estimation plot depicting the effect size for correct responses in the ‘Virtual-Real’ source monitoring task. Raw data (correct response) from sham and active session for RV task is plotted on the left ‘Y axis’. On the right ‘Y axis’, the mean of differences is plotted along with 95% CI. In this plot, the blue circles represent individual participants. The green circles with error bars represent the mean of each group with a 95% CI.

Our investigation into the 'Real-Imagine task' has yielded results that differ from those of the other tasks, both in terms of statistical analysis and practical application. There were no statistically significant differences in the observed mean sham and active tDCS scores for this task (p = 0.33; Table 1). Additionally, the small effect size, as measured by Cohen's d of 0.18, indicates a limited impact (Fig. 5).

The results suggest that our correction intervention did not elicit a statistically significant transformation in the ‘RV’ variable. However, a modest effect size implied a certain degree of alteration.

In summary, these findings provide comprehensive insights into the ramifications of our correction intervention for the variables under study. "SI" variable experienced substantial and statistically significant enhancements, marked by large effect sizes. "HI," also demonstrates a positive response, albeit with a more moderate effect size. Nonetheless, the "RV" variable remains largely unaltered by the correction intervention, failing to manifest statistical significance or a substantial effect size.

#### Adverse effects

Participants were asked to evaluate any adverse effects of the tDCS session on a Likert scale ranging from 1 to 10, where 1 indicated an absence of effects and 10 signified the highest intensity experienced. Of the 30 participants, 20% reported mild headaches; 13.3% reported scalp irritation; 10% reported fatigue; 6.7% reported itching, neck pain, and lightheadedness; and 3.3% reported a burning sensation after the session. Surprisingly, one participant reported improvement in both neck and back pain.

## Discussion

In this study, we investigated the internal source monitoring, external source monitoring, and reality monitoring abilities of a cohort of healthy participants. To modulate brain activity, we applied tDCS with the anode placed over the left STG and cathode placed over the DLPFC.

### ‘Say-imagine’ internal source monitoring error

To understand the neuronal mechanisms underlying internal source monitoring, the landscape has been characterized by notable complexity and intriguing variations in findings. Previous studies have generated somewhat contradictory results, with some shedding light on the modulation of internal source monitoring in patients with schizophrenia via repetitive transcranial magnetic stimulation^31^.

The results of the current study showed a significant difference between the sham and active tDCS sessions for the ‘SI’ task (p < 0.001) with a substantial effect size as revealed by Cohen’s d value of 0.97. In a closely related investigation, researchers discovered that tDCS improved internal source monitoring without influencing reality monitoring. It is worth noting that their study did not incorporate distractor words, a key element present in our research. The absence of such a challenge in their task might have masked potential differences that were present in our study^25^. Other studies have observed no significant effects in healthy individuals when stimulating the STG with anodal tDCS^20^. In contrast, our current study provides a fresh perspective by demonstrating a clear and distinct modulation of internal source-monitoring effects using anodal and cathodal DLPFC montages.

To reconcile these seemingly disparate outcomes, it is imperative to consider the following. First, there was no pronounced excitatory or inhibitory effect beneath the electrodes placed over the STG/DLPFC region when employing a control (sham tDCS) ipsilateral frontotemporal montage. Second, the electrical field generated by this montage was the most intense in the region between the two electrodes, precisely over the left central sulcus/Broca's area.

These findings provide a compelling explanation for the observed changes in internal source monitoring in our study. Targeted stimulation of Broca's area, which is known for its role in language processing and speech production^32^, likely played a central role. This region facilitates the translation of neural word representations into speech gestures, potentially enhancing the participants' ability to differentiate between imagined and spoken words.

Our study outcomes may also explain why previous tDCS studies targeting different brain regions or using distinct electrode placements failed to impact internal source monitoring in healthy individuals^20,22^. These observations underscore the sensitivity of tDCS outcomes to electrode placement and configuration, highlighting the complexity of brain-stimulation techniques and their effects on cognitive processes.

### ‘Hear-imagine’ external source monitoring error

Most studies have reported that the HI external source monitoring error is not affected by the STG prefrontal tDCS montage. The lack of a significant correlation between source inversions and basic auditory processing suggests that auditory disruption is more likely to be associated with reduced accuracy in recognizing the source of auditory information, rather than a specific error in attributing it to an external or internal origin. However, for the ‘Hear-Imagine’ task in the present study, a statistically significant difference between the sham and active tDCS sessions were observed that practically gave a moderate impact as indicated by Cohen’s d value of 0.44.

Several studies have noted a decrease in the severity of auditory hallucinations alongside enhancements in auditory information processing attributed to the adaptive neuroplastic effects of transcranial direct current stimulation (tDCS)^33^. This beneficial effect of tDCS may be associated with its capacity to modulate neuroplasticity, potentially by regulating GABA-mediated inhibition^8,34^. Correcting GABA deficits has been suggested as a potential mechanism responsible for tDCS's effectiveness in reducing hallucinations among individuals with schizophrenia^11,35^. These findings underscore the potential of tDCS as a valuable therapeutic approach for schizophrenia. Moreover, Broca's area's involvement in auditory corollary discharge, a process that helps us distinguish our own speech from external sounds, offers additional insights^36^. This mechanism prepares the auditory cortex for self-generated speech sounds by transmitting motor commands from speech production areas to the auditory cortex, thereby reducing the auditory cortex's response to self-generated sounds. Given our study's focus on stimulating Broca's area, it is plausible that tDCS influenced this corollary discharge process, potentially reducing source misattribution.

### ‘Real-virtual’ auditory source monitoring error

The results for the ‘Real-Virtual’ task in the current study did not reveal any significant differences between the mean sham and active tDCS scores.

The ‘real virtual’ auditory source monitoring memory task aimed to distinguish between the sources of sound in a short-term memory context. As both sources were external, they primarily functioned as short-term memory tasks. The main goal was to gain a deeper understanding of the test and cognitive processes and to investigate the potential relationship between memory and the hippocampus. While it is well established that the hippocampus is associated with memory^37^, recent research has suggested its involvement in auditory processing^38^.

Broca's area, on the other hand, is primarily associated with language function. The question arose as to whether there could be a connection between Broca's area and the hippocampus^39^, influencing memory or auditory processing. Although the task was disguised as a source monitoring error test, it essentially sought to explore associations between memory and these brain regions.

The hippocampus is a deep brain structure, and as such, is not directly stimulated by the anodal STG and cathodal DLPFC montage of tDCS; thus, its activity remains unaffected. However, as no significant differences were observed in the task, it can be concluded that the chosen montage was ineffective in modulating hippocampal activity in the context of this experiment.

### Adverse effects

The safety, comfort, and acceptance of tDCS has sparked interest in its therapeutic potential. Importantly, tDCS has been shown to be safe without severe side effects. The most common side effects reported were itching (70%), followed by a burning sensation (40%), headache (40%), tingling (30%), sleepiness (20%), mild difficulty concentrating, fatigue, skin redness, and dizziness (10%). In our study, we observed only mild adverse effects, and no severe adverse effects were observed. The quantum of adverse effects was lower than that reported in the safety profile of tDCS^40^. These results confirm that tDCS is a safe method for neuromodulation, with exciting potential for future use.

### Limitations of the study

While this study sheds light on the potential therapeutic application of tDCS in mitigating source-monitoring deficits in schizophrenia, it is crucial to recognize and address several limitations inherent in this research, the utilization of a single-session intervention prompts concerns regarding the sustainability and durability of the observed effects over time when used as a new treatment for hallucinations, extrapolating errors in source-monitoring tasks to hallucinations in individuals with schizophrenia implies a connection. However, it is essential to acknowledge that the underlying neural mechanisms may differ between induced source monitoring errors and those influenced by the disease itself, the exclusive focus on healthy individuals rather than those diagnosed with schizophrenia creates a potential gap in the clinical relevance of the findings, and similar studies in patients with schizophrenia should be designed, concerns related to blinding integrity in tDCS studies pose potential challenges, as patients may discern the current flow, and using different montages as a blinding method can yield unpredictable results. One of the limitations of the study was that during the administration of both sham and active tDCS, the patient was made to sit and was shown a relaxation video with audio. This was intended to relieve anxiety and minimize background stress and intrusive thoughts. However, this approach might also be a limitation, as auditory and visual stimulation of any kind could potentially alter brain plasticity during tDCS, thereby confounding the results. However, these limitations also present opportunities for improvement in future studies, allowing for the design of research that specifically addresses these issues.

## Conclusion

In conclusion, this study sheds light on the neural mechanisms underlying source monitoring and the promising potential of tDCS for therapeutic purposes. By delving into the effects of tDCS on source monitoring errors, particularly in internal tasks, this study highlights the impact of stimulating the left STG and DLPFC regions. This study additionally highlights the critical role of accurately placing and configuring electrodes when considering the efficacy of tDCS treatments. Furthermore, this study helps bridge the existing knowledge gaps surrounding the hypertemporal/hypofrontal model, laying the groundwork for further exploration of the specific neural mechanisms impacted by tDCS in relation to source monitoring.

The results highlighted considerable improvements in internal and external source-monitoring tasks. However, this study recognized potential constraints in resolving mistakes in actual source monitoring. Notably, discrepancies in task-specific reactions emphasize the need for fine-tuning and tailoring tDCS intervention. Furthermore, future research should investigate different brain regions, ideal stimulation techniques, and individual characteristics to optimize the accuracy and effectiveness of tDCS in addressing source-monitoring impairments in individuals with schizophrenia.

### Supplementary Information

Supplementary Information 1.

Supplementary Information 2.

Supplementary Information 3.

## Data Availability

The datasets generated during and/or analysed during the current study are available from the corresponding author on reasonable request.

## Abbreviations

tDCS: Transcranial direct current stimulation
STG: Superior temporal gyrus
DLPFC: Dorsolateral prefrontal cortex
AVH: Auditory verbal hallucinations
HI: Hear-imagine
SI: Say-imagine
RV: Real-virtual
CI: Confidence interval
