# Supplementary material for: Innovative strategies for managing hallucinations by exploring effects of tDCS on source monitoring abilities
Source: Sci Rep. 2024 Jul 17;14:16569. doi: 10.1038/s41598-024-67279-0 (PMC11254933; doi:10.1038/s41598-024-67279-0)
Supplement: Supplementary file 1 — Supplementary Information 1. [file 41598_2024_67279_MOESM1_ESM.docx]

## SUPPLEMENT A: tDCS screening questionnaire

It is important that you answer all the following questions truthfully.

If any of the questions/terms on this form are unclear or if you are unsure how to answer them, please do not hesitate to ask the researcher of the study.

|  | **Yes** | **No** |
| --- | --- | --- |
| Are you under the influence of any drug or alcohol. |  |  |
| Have you ever had a seizure? |  |  |
| Does any of your First degree relative have epilepsy? |  |  |
| Have you ever had a head injury resulting in a loss of consciousness that has required further investigation (including neurosurgery)? |  |  |
| Do you suffer from migraines? |  |  |
| Do you currently have a medical diagnosis of a psychological or neurological condition? |  |  |
| Do you have any metal in your head (outside of the mouth) such as shrapnel or surgical clips? |  |  |
| Do you have any implanted devices (e.g. cardiac pacemaker, brain stimulator)? |  |  |
| Do you have a skin condition on your scalp? (e.g. psoriasis) |  |  |
| Do you have a head wound that has not completely healed? |  |  |
| Have you ever had an adverse reaction to tDCS, or any other brain stimulation technique (e.g. TMS, tRNS)? |  |  |
| For female participants: Is there the possibility that you might be pregnant? |  |  |
| Are you currently taking any prescribed medications or are self-medicating (including reactional drug use), other than the contraceptive pill? |  |  |

The possible hazards of tDCS have been explained to me, and I understand that I can withdraw at any point for any reason, and that I do not have to disclose the reason(s) to the researcher. By signing below I acknowledge that I understand this screening form and attest to its accuracy.

| **Participant's signature** | **Researcher's signature** | **Date** |
| --- | --- | --- |
|  |  |  |
