# Supplementary material for: Innovative strategies for managing hallucinations by exploring effects of tDCS on source monitoring abilities
Source: Sci Rep. 2024 Jul 17;14:16569. doi: 10.1038/s41598-024-67279-0 (PMC11254933; doi:10.1038/s41598-024-67279-0)
Supplement: Supplementary file 2 — Supplementary Information 2. [file 41598_2024_67279_MOESM2_ESM.docx]

## SUPPLEMENT B: tDCS adverse effect questionnaire

This questionnaire will be filled in before and after receiving tDCS. Please enter a value from 1-10, ranging from absent to severe, in the ‘Rating’ space below in response to the question: “Do any of these statements currently apply to you?” It is important that you answer all questions truthfully.

1 2 3 4 5 6 7 8 9 10

Absent Severe

| Do any of these statements currently apply to you? | Rating | | Notes |
| --- | --- | --- | --- |
|  | **Before** tDCS | **After** tDCS |  |
| 1. Headache |  |  |  |
| 1. Neck pain |  |  |  |
| 1. Back pain |  |  |  |
| 1. Blurred vision |  |  |  |
| 1. Scalp irritation |  |  |  |
| 1. Tingling |  |  |  |
| 1. Itching |  |  |  |
| 1. Increased heart rate |  |  |  |
| 1. Burning sensation |  |  |  |
| 1. Hot flush |  |  |  |
| 1. Dizziness |  |  |  |
| 1. Acute mood change |  |  |  |
| 1. Fatigue |  |  |  |
| 1. Anxiety |  |  |  |
| Others: |  |  |  |
