# Supplementary material for: Innovative strategies for managing hallucinations by exploring effects of tDCS on source monitoring abilities
Source: Sci Rep. 2024 Jul 17;14:16569. doi: 10.1038/s41598-024-67279-0 (PMC11254933; doi:10.1038/s41598-024-67279-0)
Supplement: Supplementary file 3 — Supplementary Information 3. [file 41598_2024_67279_MOESM3_ESM.docx]

**SUPPLEMENT C: Summary of the published relevant studies**.

| **S. No** | **Title of the Study** | **Authors** | **Aim of the study** | **Findings of the study** |
| --- | --- | --- | --- | --- |
| 1. 1. | Fronto-temporal transcranial Direct Current Stimulation (tDCS) reduces source-monitoring deficits and auditory hallucinations in patients with schizophrenia | Mondino et al [16] | To investigate the effect of frontotemporal tDCS on source monitoring processes in patients with treatment-resistant auditory verbal hallucinations (AVH). | Auditory verbal hallucinations were reduced at tDCS in patients with schizophrenia. |
| 1. 2. | Examining transcranial direct-current stimulation (tDCS) as a treatment for hallucinations in schizophrenia | Brunelin et al [19] | The effectiveness of tDCS in reducing the severity of auditory verbal hallucinations and negative symptoms. | Auditory verbal hallucinations were reduced by tDCS compared to sham stimulation. The authors also observed improvements in other symptoms, particularly in the negative symptoms. |
| 1. 3. | Anodal tDCS targeting the left temporo-parietal junction (TPJ) disrupts verbal reality-monitoring | Mondino et al [20] | To investigate the causal role of the left temporoparietal and prefrontal regions in source monitoring using transcranial direct current stimulation (tDCS) | Active tDCS over the left TPJ decreased reality monitoring performance but did not affect internal source monitoring performance. Active tDCS over the left PFC did not influence performance in either task. In both experiments, the reference electrode was placed over the right occipital region. |
| 1. 4. | Investigating the roles of medial prefrontal and superior temporal cortex in source monitoring | Moseley et al [21] | To study the effects of transcranial direct current stimulation (tDCS) on reality monitoring by modulating the excitability of the medial prefrontal cortex and superior temporal cortex. | tDCS applied to the superior temporal or medial prefrontal regions did not affect reality monitoring performance. This finding also questions the models linking reality monitoring to the therapeutic effects of tDCS on auditory verbal hallucinations. |
| 1. 5. | Transcranial direct current stimulation (tDCS) enhances internal source monitoring abilities in healthy participants | Kusztrits, et al [25] | The aim of the study was to directly test the hypertemporal/hypofrontal model for source monitoring abilities by placing the anode over the left STG and the cathode over the left DLPFC in healthy individuals | tDCS over prefrontal and temporoparietal areas affects source monitoring in healthy participants. |
| 1. 6. | Low frequency repetitive transcranial magnetic stimulation improves source monitoring deficit in hallucinating patients with schizophrenia. | Brunelin, et al [31] | To investigate the relationship between improvements in auditory hallucinations and source monitoring performance after rTMS treatment at the left temporoparietal cortex | Active rTMS significantly improved auditory hallucinations compared to the sham treatment. |
| 1. 7. | Neural basis of tDCS effects on auditory verbal hallucinations in schizophrenia: a case report evidence for cortical neuroplasticity modulation | Nawani et al [33] | To explore the effect of transcranial direct current stimulation (tDCS) on auditory hallucinations that are nonresponsive or minimally responsive to antipsychotic treatment in schizophrenia. | tDCS helps in improving treatment-resistant auditory hallucinations in a patient with schizophrenia. |
